# Parallel in time dynamics with quantum annealers

**DOI:** 10.1038/s41598-020-70017-x

**Published:** 2020-08-11

**Authors:** Konrad Jałowiecki, Andrzej Więckowski, Piotr Gawron, Bartłomiej Gardas

**Affiliations:** 1 Institute of Physics, University of Silesia, 75 Pułku Piechoty 1, 41-500 Chorzów, Poland; 2 Department of Theoretical Physics, Faculty of Fundamental Problems of Technology, Wrocław University of Science and Technology, 50-370 Wrocław, Poland; 3 Institute of Theoretical and Applied Informatics, Polish Academy of Sciences, Bałtycka 5, 44-100 Gliwice, Poland; 4 AstroCeNT, Nicolaus Copernicus Astronomical Center, Polish Academy of Sciences, ul. Rektorska 4, 00-614 Warsaw, Poland; 5 Institute of Physics, Jagiellonian University, Łojasiewicza 11, 30-348 Kraków, Poland

**Keywords:** Quantum physics, Quantum simulation

## Abstract

Recent years have witnessed an unprecedented increase in experiments and hybrid simulations involving quantum computers. In particular, quantum annealers. There exist a plethora of algorithms promising to outperform classical computers in the near-term future. Here, we propose a parallel in time approach to simulate dynamical systems designed to be executed already on present-day quantum annealers. In essence, purely classical methods for solving dynamics systems are serial. Therefore, their parallelization is substantially limited. In the presented approach, however, the time evolution is rephrased as a ground-state search of a classical Ising model. Such a problem is solved intrinsically in parallel by quantum computers. The main idea is exemplified by simulating the Rabi oscillations generated by a two-level quantum system (i.e. qubit) experimentally.

## Introduction

It is needless to say that simulating dynamical systems with near-term quantum technology poses one of the most difficult and technologically challenging endeavor^1^. Various computations of certain aspects of many-body quantum physics can already be assisted by the existing hardware^2–4^. For instance, recent experiments have demonstrated that quantum annealers^5^ can be turned into neural networks that can learn the ground state energy of a physical system^6^. A similar task can also be accomplished with fewer qubits using quantum gates^7, 8^.

The aforementioned examples characterize static processes where there is no real-time dynamics being simulated *directly*. Noticeably, near-term quantum annealers *do* simulate quantum annealing, which is a time-dependent phenomenon. However, the optimization problem itself, i.e., the one to be solved by the annealer, exhibits no time dependence^9^. Thus, following the time evolution, even of a single qubit on a quantum annealer is a challenging task for the current technology. This should, nonetheless, be possible at least in principle. Indeed, a time-dependent quantum problem can be (re)formulated as a static one, defined on an appropriately enlarged Hilbert space^10^. This is realized using the Feynman’s clock operator^11, 12^.

This observation naturally encapsulates a family of powerful algorithms referred to as *parallel in time* or *parareal* methods, often invoked to simulate the system’s dynamics on heterogeneous classical hardware^13, 14^. The latter techniques effectively take advantage of the fact that a part of the evolution can be distributed and carried out in parallel. Nevertheless, with such an approach, one can never reach full parallelism on any classical hardware (of the Turing type) due to the communication bottlenecks^15^. Nonetheless, these limitations do *not* apply to the quantum hardware. Quite the contrary, quantum computers operate in parallel and any algorithm (cf. Refs.^16–18^) they execute needs to be carefully designed from scratch to utilize their intrinsic parallelism fully.

As a proof of concept, in this article, we demonstrate how present-day quantum annealers may be programmed to simulate dynamical systems in parallel (due to their noisiness only in the specific regime of the problem’s parameters). In particular, we determine the time evolution of a single qubit (Rabi oscillations) solely from experiments conducted on the newest D-Wave 2000Q quantum chip^19–22^. At the same time, due to the underlying connectivity (all-to-all) and the extensive amount of qubits it requires, the proposed algorithm constitutes a natural test which can determine the usefulness of various annealing technology realized by e.g. the Floquet annealer^23^, the large-scale (photonic^24^) Ising machines^25–28^, and the Fujitsu digital annealer^29^ in simulating physical systems.

## Parallel in time dynamics

Consider a dynamical system (e.g. a quantum system isolated from its environment^30^) whose behavior can be described by a *L* dimensional and possibly time-dependent, *K*amiltonian *K*(*t*) (“*K*amiltonian” refers to a Hamiltonian-like operator, *K*^31^.). The system dynamics is encoded, at all times, in a (quantum) state, $| \psi (t) \rangle$, whose evolution is governed by a Schrödinger like equation^32^,

1 $$\begin{aligned} \frac{\partial | \psi (t) \rangle }{\partial t} = K(t)| \psi (t) \rangle . \end{aligned}$$

This first order differential equation admits a unique solution $| \psi (t) \rangle :=U(t,t_0)| \psi (t_0) \rangle$, where

2 $$\begin{aligned} U(t,t_0) = {{\mathscr {T}}} \exp \left( \int _{t_0}^{t} K(\tau ) d\tau \right) , \end{aligned}$$

propagates an arbitrary initial state, $| \psi (t_0) \rangle$, from $t_0$ to $t\ge t_0$ whereas ${{\mathscr {T}}}$ denotes the time-ordering operator^33^. Such an ordering can be omitted whenever $[K(t), K(t^{\prime })]=0$. In particular, for time independent systems, $\partial _t K(t)=0$. Furthermore, when $K(t)=-iH(t)/\hbar$ where $H(t)^{\dagger }=H(t)$ is a Hamiltonian, the evolution operator (2) is unitary and the dynamics (1) is norm preserving and reversible, i.e. $U(t,t^{\prime })^{\dagger }=U(t,t^{\prime })^{-1}=U(t^{\prime },t)$.

To solve Eq. (1), one usually discretizies the time interval $[t_0,t]$ selecting *N* distinct moments, i.e. $t:=t_{N-1}> \dots> t_{n+1}> t_{n}> \dots > t_{0}$. The dynamics can then be formulated as a sequence of unitary gates,

3 $$\begin{aligned} U(t,t_0) = U_{N-1} \cdots U_{n+1}U_{n} \cdots U_0, \end{aligned}$$

acting on an initial state. Note, each $U_n:=U(t_{n+1},t_n)$ can also be formally expressed using Eq. (2). Practically, however, for small time steps, all gates $U_n$ are approximated using variety of methods^32^. Those include exact diagonalization for small system^34^, Suzuki–Trotter decomposition^35^, commutator-free expansion^36^ or sophisticated tensor networks techniques^37^.

**Figure 1:**
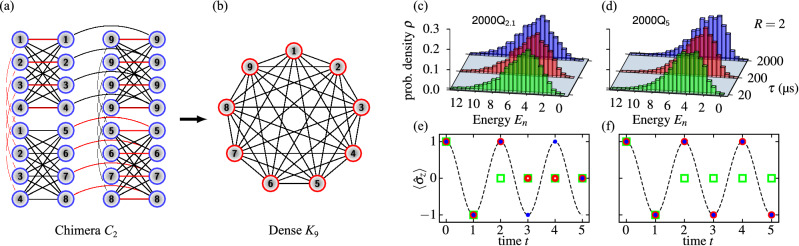
(**a**) An example of a sparse chimera graph [here $C_{2}$ (e.g., $2\times 2\times 8$) consisting of $2 \cdot 2 \cdot 8=32$ qubits, cf. Eq. (12)] and (**b**) the 9 qubits complete graph $K_9$ embedded on $C_2$. Certain interactions on the chimera graph (marked as red) effectively “glue” physical qubits, ${{\hat{\sigma }}}^z_j$, to form logical variables, $q_i^{\alpha }$. (**c**)–(**f**) Rabi oscillations simulated on two generations of D-Wave quantum annealers. (**c**, **d**) the distribution of energy outputted by the annealers for different annealing times $\tau$. The two instances were generated from Eq. (9) where $R=2$ bits of precision was assumed. The total number of variables in the corresponding QUBO was $|V|=168$. Probability distributions are constructed from $10^4$ samples retrieved from quantum annealers. (**e**, **f**) the evolution in time of the spin *z*-component of a two level system (13), $\omega =\pi /2$. The annealing time (green open square—20, red open circle—200, blue dots—2000) is measured in microseconds.

The latter equation provides a starting point for various sequential numerical schemes for solving differential equations on classical computers^38^. In principle, however, those gates could also be realized on a quantum computer, which could then resolve the unitary dynamics efficiently^32^. Unfortunately, current quantum hardware does not allow for such gates to be constructed yet. Nevertheless, the underlying idea behind decomposition (3) can be harnessed to formulate an optimization problem that can be solved by present-day quantum annealers^4^. This is the main idea we put forward in this work.

Indeed, consider a superposition of quantum states in different moments of time $t_n$,

4 $$\begin{aligned} | \Psi \rangle = \sum _{n=0}^{N-1} | t_n \rangle \otimes | \psi (t_n) \rangle , \end{aligned}$$

where the *clock* states are orthonormal, $\langle t_n | t_m \rangle =\delta _{nm}$. With the corresponding clock operator,

5 $$\begin{aligned} {{\mathscr {C}}} = \sum _{n=0}^{N-2} \big ( | t_{n+1} \rangle \langle t_{n+1} | \otimes I - | t_{n+1} \rangle \langle t_n | \otimes U_n + \text {h.c.} \big ), \end{aligned}$$

one obtains ${{\mathscr {C}}} | \Psi \rangle =0| \Psi \rangle$. Thus, $| \Psi \rangle$ is the ground state of ${{\mathscr {C}}}$. Obviously, this state is not unique since we have specified neither initial nor boundary condition. However, introducing a penalty, say ${{\mathscr {C}}}_{0}$, allows one to provide additional constrains. In particular, specifying that ${{\mathscr {C}}}_0=| t_0 \rangle \langle t_0 |\otimes (I-| \psi _0 \rangle \langle \psi _0 |)$, the following linear system

6 $$\begin{aligned} {{\mathscr {A}}} | \Psi \rangle = | t_0 \rangle \otimes | \psi _0 \rangle , \quad {{\mathscr {A}}}={{\mathscr {C}}} + | t_0 \rangle \langle t_0 | \otimes I, \end{aligned}$$

encodes Eq. (1) subjected to $| \psi (t_0) \rangle =| \psi _0 \rangle$. For hermitian systems, the above complex linear system of $N\times L$ equations expresses the reversible dynamics of the system in terms of a sequence of unitary gates (3). The hermitian clock operator can also be derived from e.g. time-embedded discrete variational principle (that is, the principle of least action)^10^. The idea can be further extended to open quantum systems^39^.

To solve the dynamics expressed in Eq. (6) on a quantum annealer one needs to formulate it as an optimization problem^12, 40, 41^. Moreover, such an optimization needs to be encoded via the Ising spin-glass Hamiltonian^5^ (or QUBO^42^) defined on a particular *sparse* graph called chimera^43^ (or pegasus^44^). We stress that these particular graphs are specific for the D-Wave hardware, and other architectures allow for a different connectivity between qubits. For instance, all-to-all (complete graph) in case of the (classical) Fujitsu Digital annealer^29^. Furthermore, at least complex fixed-point arithmetic is also required to express quantum states in consecutive moments of time^45^. Here, we incorporate a strategy introduced only recently in Ref.^46^, cf. also Ref.^45^ for real matrices. To this end, we employ a natural correspondence between complex numbers and real $2 \times 2$ matrices, namely $a+bi \mapsto a {\hat{I}}+ib{\hat{\sigma }}_y$, to represent ${{\mathscr {A}}}$ using only real entries.

We further rely on a straightforward observation that the solution to Eq. (6), expanded in the standard basis as $| \mathbf{x } \rangle =\sum x_i| i \rangle$, also minimizes the following functional $h({\mathbf{x }})=\Vert {{\mathscr {A}}}| \mathbf{x } \rangle -| \Phi \rangle \Vert ^2$ and *vice versa*. That is, a global minimum of *h*, i.e. ${\mathbf{x }}_0$ is a solution (6) as $h({\mathbf{x }}_0)=0$. Moreover, when the simulated system is hermitian then ${{\mathscr {A}}}$ is positive definite. Therefore, ${\mathbf{x }}_0$ is also a minimum of

7 $$\begin{aligned} f({\mathbf{x }})=\frac{1}{2}\langle \mathbf{x } |{{\mathscr {A}}}| \mathbf{x } \rangle - \langle \mathbf{x } | \Phi \rangle \quad \text {as} \quad \nabla f({\mathbf{x }}) = {{\mathscr {A}}}| \mathbf{x } \rangle -| \Phi \rangle , \quad \text {and} \quad \nabla ^2 f({\mathbf{x }})={{\mathscr {A}}}>0. \end{aligned}$$

Henceforward, we consider only hermitian systems and focus exclusively on the latter equation (This is mostly due to the technical limitations (e.g. coupling’s precision) of the current annealing technology.).

Since variables $x_i$ are real, the objective functions $f({\mathbf{x }})$ can *not* be programmed directly to be optimized on a quantum annealer. Nevertheless, one can obtain the so called fixed-point representation for each $x_i$ as a linear combination of *binary* variables $q_i^{\alpha }$^45^

8 $$\begin{aligned} x_i = 2^D \left( 2 \sum _{\alpha =0}^{R-1}2^{-\alpha }q_i^{\alpha } -1\right) . \end{aligned}$$

The above correspondence is constructed with the assumption that *R* bits of binary representation are used for every real number in the solution vector. In our approach, the order of magnitude of the solution’s coefficients is also assumed, i.e. $x_i \in [-2^D, 2^D]$ for a fixed $D \in {\mathbb {N}}$.

Therefore, the minimization problem to be solved on a quantum annealer can finally be formulated as

9 $$\begin{aligned} f({\mathbf{q }}) = \sum _{i,\alpha } a_i^{\alpha } q_i^r + \sum _{i,j,\alpha ,\beta } b_{ij}^{\alpha \beta } q_i^{\alpha } q_j^{\beta } + f_0, \end{aligned}$$

where

10 $$\begin{aligned}&b_{ij}^{\alpha \beta } = {{\mathscr {A}}}_{ij} 2^{1-\alpha -\beta +2D} \quad \text {and} \quad a_i^\alpha = \left( 2^{-\alpha +D}{{\mathscr {A}}}_{ii} - 2^D\sum _{j}{{\mathscr {A}}}_{ij}- \phi _i\right) 2^{1-\alpha +D}, \nonumber \\&f_0 = 2^D\left( 2^{D-1}\sum _{ij}{{\mathscr {A}}}_{ij}+\sum _i \phi _i\right) . \end{aligned}$$

The constant energy contribution, $f_0$, can be omitted as both $f({\mathbf{q }})$ and $f({\mathbf{q }})-f_0$ have the same optimal solution ${\mathbf{q }}_0$. Since $f({\mathbf{q }}_0)=0$, one can easily asses the quality of the solution found by any heuristic approach.

For small *N*, QUBO (9) is defined on a complete graph [cf. Fig. 1b] with $|{{\mathscr {V}}}|=R\times N \times (2L)$ vertices. In contrast, when $N\gg 2$ the number of edges is equal to the number of nonzero elements of ${{\mathscr {A}}}$ which is sparse. Currently, the biggest complete graph that can be embedded on the 2000Q chip has $|{{\mathscr {V}}}|=65$ vertices ($|{{\mathscr {V}}}|=180$ for the Pegasus topology^47^), cf. Fig. 1. It is worth mentioning that classical solvers (hardware-based or otherwise) usually offer better connectivity and thus can realize much denser graphs without the need for embedding. For example, the so-called coherent Ising machines (among others) can incorporate complete graphs consisting of the order of $10^3$ vertices^26^. Therefore, QUBO generated from the dynamics provide a natural “stress” test for those machines which can asses their usefulness in simulating physics.

## Quantum annealing

Adiabatic quantum computing can be seen as an alternative paradigm of computation^5^. Essentially, it is equivalent to the gate model of quantum computation that uses logical gates operating on quantum states to implement quantum algorithms^32^. The main idea is based on the quantum adiabatic theorem^48^. When a system starting from its ground state is driven slowly enough, it has time to adjust to any change, and thus it can remain in the ground state during the entire evolution.

Assume a quantum system is prepared in the ground state of an initial (“simple”) Hamiltonian ${{\mathscr {H}}}_0$. Then, it will slowly evolve to the ground state of the final (“complex”) Hamiltonian ${{\mathscr {H}}}_{\mathrm{p}}$ that one can harness to encode the solution to an optimization problem. In particular, the dynamics of the current D-Wave 2000Q quantum annealer is supposed to be governed by the following time-dependent Hamiltonian (cf. Ref.^9^)

11 $$\begin{aligned} {{\mathscr {H}}}(s)/(2\pi \hbar )= -g(s) \sum _{i} {\hat{\sigma }}_{i}^x -\Delta (s) {{\mathscr {H}}}_{\mathrm{p}}, \quad s \in [0, \tau ], \end{aligned}$$

where the problem Hamiltonian ${{\mathscr {H}}}_{\mathrm{p}}$ realizes the spin-glass Ising model defined on the chimera graph, $({{\mathscr {E}}},{{\mathscr {V}}})$, specified by its edges and vertices,

12 $$\begin{aligned} {{\mathscr {H}}}_{\mathrm{p}} = \sum _{\langle i, j\rangle \in {{\mathscr {E}}}} J_{ij} {\hat{\sigma }}_i^z {\hat{\sigma }}_j^z + \sum _{i\in {{\mathscr {V}}}}h_i {\hat{\sigma }}_i^z. \end{aligned}$$

The annealing time $\tau$ varies from microseconds to milliseconds depending on the programmable schedule^9^. Typically, during the evolution *g*(*s*) varies from $g(0) \gg 0$ [i.e. all spins point in the *x*-direction] to $g(\tau )\approx 0$ whereas $\Delta (s)$ is changed from $\Delta (0)\approx 0$ to $\Delta (\tau ) \gg 0$ [i.e. ${{\mathscr {H}}}(\tau ) \sim {{\mathscr {H}}}_{\mathrm{p}}$]. Note, the Hamiltonian ${{\mathscr {H}}}_{\mathrm{p}}$ is classical in a sense that all its terms commute. Thus, its eigenstates translate directly to classical optimization variables, $q_i^{\alpha }$, which we introduced to encode the time evolution (6) as QUBO (9). The Pauli operators ${\hat{\sigma }}_i^z$, ${\hat{\sigma }}_i^x$ describe the spin degrees of freedom in the *z*- and *x*-direction respectively.

Dimensionless real couplers, $J_{ij}\in [-1,1]$, and magnetic fields, $h_i\in [-2,2]$, are programmable. In practice, the actual values of those parameters that are sent to the quantum processing unit differ from the ones specified by the user by a small amount $\delta J_{ij}$, $\delta h_i$^49^. This is due to various reasons including noise effects which we will neglect in this work (cf. Ref.^4, 50^).

Most practical optimization problems are defined on dense graphs which can be embedded onto the chimera graph^51^. There is, however, a substantial overhead that effectively limits the size of problems that can be solved with current quantum annealers. This is, nonetheless, an engineering issue that will most likely be overcome in the near future^23, 47^.

## Results

To exemplify the main idea we consider a two-level quantum system (qubit) whose Hamiltonian reads

13 $$\begin{aligned} H = \omega {\hat{\sigma }}_y, \end{aligned}$$

where ${\hat{\sigma }}_y$ is the Pauli spin matrix in the *y*-direction. For the sake of simplicity, we further set $\omega =\pi /2$. Moreover, due to the limited number of qubits and sparse connectivity of D-Wave quantum annealers, we mainly consider the system’s evolution at six distinct integer time points, starting from $| \psi _0 \rangle =|0\rangle$. This ensures that the dynamics can be captured precisely with two bits of precision per component of the state vector, thus allowing one to run experiments on the D-Wave 2000Q annealer. For the illustrative purposes we reconstruct $\langle {\hat{\sigma }}_z\rangle (t)$.

As depicted in Fig. 1, the low noise D-Wave 2000Q annealer was able to capture the dynamics faithfully [cf. Fig. 1d,f], for $\tau =200$ µs, 2000 µs. Therein, probability distributions, $\rho$, were constructed from $10^4$ anneals. There were no post-processing involved and the Boltzmann temperature, $\beta$, was set to its default value. Furthermore, to construct the dynamics (Fig. 1d,f) only the lowest energy state, reported by the device, was utilized. For this particular problem excited states (also returned by the annealer) are not, a priori useful. This experiments demonstrate an improvement in comparison to the (not that) older generation, results for which are shown in Fig. 1c and e.

**Figure 2:**
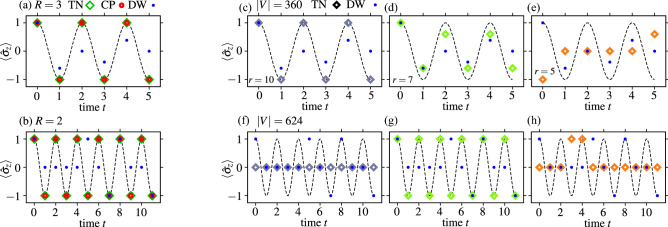
(**a**, **b**) Performance of the two state of the art heuristic algorithms: the CPLEX optimizer (CP) and a recent solver based on tensor networks (TN) in comparison to the D-Wave 2000Q quantum annealer (DW), cf. Fig 1. The corresponding QUBO instances (encoded using double numerical precision) had total of $|V|=360$ and $|V|=624$ spin variables for (**a**), and (**b**) respectively. The annealing time was set to $\tau =200$µs. The numerical precision of the solution vector is denoted as *R*. (**c**)–(**h**) Degradation of the solution quality resulting from the truncation of the problem coefficients, cf. Eq. (10), to a given numerical precision denoted as *r*. The numerical results were obtained by finding the ground state with tensor networks (TN). As a reference point, we included experimental data from the D-Wave 2000Q quantum annealer (DW). This effect is expected to be predominant for the current quantum annealing technology. It is already visible on Figs. 1c–f,  2a,b and it further increases with the increasing graph size *V*.

In contrast, results obtained from an emulation of the D-Wave output with tensor networks (cf. Ref.^52^) are presented in Fig. 2. As a reference point, we have also included solutions found by the CPLEX optimizer^53^. Both these solvers, being purely classical, exhibit superior performance in comparison to the D-Wave quantum annealers (Assuming sufficiently large precision of all ${{\mathscr {A}}}_{ij}$.). This is noticeable especially for problems that require bigger graphs resulting from higher precision—($R\ge 3$, $N=6$), cf. Fig. 2a—or extra time points ($N>6$, $R=2$), cf. Fig. 2b. Similar degradation of the solution quality with the increasing problem size has been observed, e.g., in Ref.^54, 55^ in the context of problems requiring complete graphs, cf. Fig. 1. The behavior, as mentioned above, is expected from an early stage device which is prone to errors. Their origins, however, are anything but straightforward to pinpoint precisely. In stark contrast, there is yet another source of errors that is related to the precision of $J_{ij}$, and $h_i$^56^. Those errors are believed to be predominant for the type of simulations introduced in this work. Indeed, Fig. 2c–h shows the destructive (above all *not* monotonic) effect of the limited precision—*r*, of the problem coefficients ${{\mathscr {A}}}_{ij}$—on the solution. Beyond a certain threshold, neither the D-Wave annealer nor the aforementioned classical heuristics can reproduce the dynamics (i.e., oscillations) accurately.

As a final note, we stress that the precision *R* (the only one that influences the graph size) is the number of bits required for representing the discretized continuous variables, *x* [cf. Eq. (8)], which is *different* from *r*. The latter is an inherent characteristic of the hardware which stems from the DAC quantization step (for more details cf. D-Wave’s technical notes^56^).

## Conclusions

In this article, we have proposed a parallel in time approach to simulate dynamical systems with the quantum annealing technology. While one should not expect current quantum annealers to be faster/better than classical computers, our results constitute, first and foremost, a proof of concept demonstrating how the technology can be employed to simulate the time evolution of simple (e.g. two-level) quantum systems (in certain regime of the corresponding parameters). This task is a priori difficult for the current prototypical quantum computers which are prone to errors and has been designed mostly to simulate static phenomena.

Furthermore, not only the Ising instances we have generated can be executed on the commercially available D-Wave annealers, but they can also be tested on: coherent Ising machines^24–28^, the Floquet annealer^23^, and the Fujitsu digital annealer^29^ that celebrate all-to-all connectivity. This provides a practical “stress” test for those machines which can determine their usefulness in simulating various time-dependent properties of physical systems.
